# Estimation of Neonatal Intestinal Perforation Associated with Necrotizing Enterocolitis by Machine Learning Reveals New Key Factors

**DOI:** 10.3390/ijerph15112509

**Published:** 2018-11-09

**Authors:** Claudine Irles, Gabriela González-Pérez, Sandra Carrera Muiños, Carolina Michel Macias, César Sánchez Gómez, Anahid Martínez-Zepeda, Guadalupe Cordero González, Estibalitz Laresgoiti Servitje

**Affiliations:** 1 Department of Physiology and Cellular Development, Instituto Nacional de Perinatología Isidro Espinosa de los Reyes, Mexico City 11000, Mexico; gonzalezperez.gabriela@gmail.com (G.G.-P.); kronos.et.apolo@gmail.com (C.S.G.); anahid454@gmail.com (A.M.-Z.); 2 Department of Neonatal Intensive Care, Instituto Nacional de Perinatología Isidro Espinosa de los Reyes, Mexico City 11000, Mexico; sandracarreram@hotmail.com (S.C.M.); dra.carolinamichel@gmail.com (C.M.M.); guadita69@yahoo.com.mx (G.C.G.); 3 Focus Group on Cardiovascular Medicine and Metabolomics, Escuela de Medicina ABC-ITESM, Mexico City 11000, Mexico; estibalitz.laresgoiti@itesm.mx

**Keywords:** prematurity, surgical necrotizing enterocolitis, computer simulation

## Abstract

Intestinal perforation (IP) associated with necrotizing enterocolitis (NEC) is one of the leading causes of mortality in premature neonates; with major nutritional and neurodevelopmental sequelae. Since predicting which neonates will develop perforation is still challenging; clinicians might benefit considerably with an early diagnosis tool and the identification of critical factors. The aim of this study was to forecast IP related to NEC and to investigate the predictive quality of variables; based on a machine learning-based technique. The Back-propagation neural network was used to train and test the models with a dataset constructed from medical records of the NICU; with birth and hospitalization maternal and neonatal clinical; feeding and laboratory parameters; as input variables. The outcome of the models was diagnosis: (1) IP associated with NEC; (2) NEC or (3) control (neither IP nor NEC). Models accurately estimated IP with good performances; the regression coefficients between the experimental and predicted data were *R*^2^ > 0.97. Critical variables for IP prediction were identified: neonatal platelets and neutrophils; orotracheal intubation; birth weight; sex; arterial blood gas parameters (pCO_2_ and HCO_3_); gestational age; use of fortifier; patent ductus arteriosus; maternal age and maternal morbidity. These models may allow quality improvement in medical practice.

## 1. Introduction

Intestinal perforation (IP) associated with necrotizing enterocolitis (NEC), also known as surgical NEC, is one of the leading causes of death in preterm infants, with up to 30–50% mortality compared with only 21% in those medically treated for NEC [1,2,3,4,5]. Global prevalence of intestinal perforation due to NEC is 27–52% [6] with 90% of cases in premature infants [7,8,9,10,11]. Furthermore, surgical NEC leads to an increase in the length of stay beyond expected for prematurity alone, along with a considerable increase in costs [12,13]. Survivors are affected by significant complications and comorbidities, including sepsis, short bowel syndrome, adhesions, cholestasis and impaired neurodevelopment [14,15,16,17]. These evidences highlight the burden of intestinal perforation by NEC and the need for a tool which allows the clinician to estimate surgical NEC and to guide the medical management accordingly. NEC may present with abdominal distention, emesis, bloody stools, temperature instability, apnea and bradycardic spells, abdominal wall erythema or edema, pneumatosis, portal venous gas and/or pneumoperitoneum [18], but few of them with prognosis value [18,19]. As NEC has a broad spectrum of disease severity and clinical presentation, we aimed to forecast infant intestinal perforation in order to increase awareness and early institutional management [18].

Despite the existence of prediction tools and early recognition of NEC, it remains challenging to estimate intestinal perforation, as it results from intricate interrelationship between multiple factors, being difficult to predict in individual cases. In this regard, machine learning models are able to predict outcomes from complex non-linear interactions [20], which are difficult to obtain using conventional linear statistical analyses [21,22]. Specifically, artificial neural networks (ANN) “learn” from training data and allow a robust and more accurate estimation of complex patterns. Such tools are currently being used in medical decision support systems [23,24]. Therefore, the aim of this study was to develop models to estimate intestinal perforation in premature neonates with NEC, based on ANN, evaluating maternal and patients’ multifaceted variables, at birth and during hospitalization. Secondly, to identify key maternal and neonatal factors (“risk factors”), we performed a sensitivity analysis which determines the impact of the variables in predicting the diagnosis. ANN were trained with a back-propagation algorithm (BPNN) [25] without *a priori* assumptions regarding the relationship between variables. The short-elapsed time needed to calculate the IP diagnosis makes the online application of such models possible. We have previously used BPNN in a biomedical context, with accurate results for the prediction of neonatal and maternal biochemical blood parameters [26,27]. Such models will be a key contribution for prevention, early diagnosis, follow-up and quality improvement in medical practice in a clinical setting.

## 2. Materials and Methods

### 2.1. Study Design and Ethical Approvals

This was an observational retrospective study. Data were obtained from patient records hospitalized in the Neonatal Intensive Care Unit (NICU) of a tertiary care hospital from January 2015 to August 2017. Informed consent was not required (protocol approved by the Institutional Research and Ethics Boards of the Instituto Nacional de Perinatología Isidro Espinosa de los Reyes, certificate number: 2017-2-65).

This work was designed to forecast IP related to NEC and to investigate the predictive quality of variables. For this purpose, we developed two estimation models for intestinal perforation associated with NEC: (a) an ANN model at birth and (b) an ANN model at birth and during hospitalization. Three groups of neonates were compared: (1) control group without NEC nor intestinal perforation but with similar gestational age (*N* = 27), (2) NEC group (according to Bell’s staging criteria) (*N* = 23) and (3) Intestinal perforation associated with NEC group (Bell’s Stage IIIB) (*N* = 26). We excluded 15 cases with incomplete clinical information as well as spontaneous or not associated with NEC intestinal perforation, as well as digestive tract malformations. Control neonates were not stage I NEC (or any NEC stage). We carefully collected a balanced dataset with a similar number of samples per group.

Diagnosis of NEC and intestinal perforation associated with NEC was defined according to modified Bell’s staging criteria [28] modified by Walsh [29]. NEC patients included those presenting bedside KUB radiographic findings described in stages IIA, IIB, and IIIA as follows: Ileus with dilated bowel loops and focal pneumatosis, or widespread pneumatosis, or portal venous gas with or without ascites, without free air. Patients with intestinal perforation related to NEC included those presenting radiographic findings described in stage IIIB as follows: Pneumoperitoneum.

### 2.2. Dataset

Taking into account variables already reported in the literature, as well as others proposed during meetings with the clinical staff, we chose 113 variables that included maternal and neonatal data recorded at birth and during hospitalization. Within these, we collected maternal and neonatal clinical/demographic data (maternal age, maternal morbidity, gestational age, birth weight), diagnosis, oxygen therapy at birth, enteral feeding, laboratory, and clinical findings. Routine laboratory tests are performed for all premature neonates <35 weeks and, within the first 24 h for closer monitoring, and included arterial pH, blood gases and hematologic data. Maternal obesity was defined as a Body Mass Index (BMI, calculated as weight (kg)/height (m^2^) greater or equal to 30 (World Health Organization). Chorioamnionitis was defined as an acute inflammation or infection of any combination of fetal membranes, amniotic fluid, decidua and chorion of the placenta. Numerical data were expressed by a number, whereas absence or presence (no or yes) was expressed as 0 or 1, respectively. Differences in numerical variables between groups were analyzed by ANOVA, and categorical variables were analyzed by Pearson’s Chi-square test (SPSS version 22, IBM, Armonk, NY, USA). Twenty-three variables were chosen for the IP associated with NEC model at birth, while 35 parameters were selected for the IP associated with NEC model at birth and during hospitalization (data were taken 24 h before intestinal perforation diagnosis). The anthropometric/clinical maternal and neonatal characteristics are depicted in Table 1 and Table 2, respectively.

### 2.3. ANN (Learning, Testing, and Validation)

The architecture of ANN models comprised three layers of neurons (nodes) connected together: an input layer (parameters predicting the outcome), a hidden layer (activation transfer functions) and an output layer: the prediction of diagnosis, either (1) no NEC nor IP, (2) NEC or (3) IP associated with NEC. The database (*N* = 76 neonates) was randomly divided into training (80%) and testing, validation (20%). The input variables were normalized in the range of 0.1 to 0.9, as previously described [26], in order to prevent within-patient differences in variation and amplitude among variables, and the output variable was not normalized. The Back-propagation neural network (BPNN) was used to train and test ANN models using the Levenberg-Marquardt algorithm [30], as previously explained [26,27]. Briefly, in the hidden layer, one to <5 neurons were applied until the Root Mean Square Error (RMSE) between the experimental data (Target) and predicted values (network) was <10^−12^, as well as validation of the model by the slope and intercept statistical test (see Section 2.3.3) and avoiding overfitting (performance evaluation of the model through training, testing, and validation).

#### 2.3.1. IP ANN Model at Birth

Twenty-three input variables were chosen from the entire database with the following maternal parameters: maternal age (MA), maternal morbidity (MM, see list in Appendix A), chorioamnionitis (C, y/n), prenatal antibiotic (PA, y/n), number of offsprings, Premature rupture of membranes (PRM, y/n), and neonatal variables at birth: gestational age (GA), oxygen therapy at birth (y/n): indirect oxygen (IO, y/n) T-piece (T-P, y/n), Continuous positive airway pressure (CPAP, y/n), positive pressure ventilation cycles (PPV, y/n), orotracheal intubation (OTI, y/n), birth weight (BW), sex (S), arterial pH (pH), blood gas: CO_2_ (pCO_2_), HCO_3_, base deficit (BD), diastolic arterial blood pressure (DBP), number of total leukocytes (L), neutrophils (N), platelets (P), and catheter location (CL). Input variables range is shown in Table 3.

#### 2.3.2. IP ANN Model at Birth and during Hospitalization

The 35 input variables were maternal and neonatal parameters at birth as well as during hospitalization: maternal age (MA), maternal morbidity (MM, see list in Appendix A), chorioamnionitis (C, y/n), prenatal antibiotic (PA, y/n), Premature rupture of membranes (PRM, y/n), maternal infection (MI, y/n); neonatal variables at birth: Intrauterine Growth Restriction (IUGR, y/n), gestational age (GA), oxygen therapy at birth (y/n): indirect oxygen (IO), T-piece (TP), Continuous positive airway pressure (CPAP), orotracheal intubation (OTI), birth weight (BW), sex (S), laboratory findings at birth: arterial pH (pH), blood gas: (pCO2), (HCO3), base difference (BD), number of total leukocytes (L), neutrophils (N), platelets (P); first day of oral feeding (OF), use of human milk 24 h before diagnosis of IP (HM, y/n); use of formula 24 h before diagnosis of IP (F, y/n); use of fortifier 24 h before diagnosis of IP (FF, y/n); presence of apnea (A, y/n); presence of gastric residuals 24 h before diagnosis of IP (GR, y/n); number of antibiotic schemes (Ant, y/n); presence of patent ductus arteriosus (PDA, y/n); number of blood transfusions (T, y/n); presence of hypotension (H, y/n); early sepsis (ES, y/n); late sepsis (LS, y/n) and catheter location (CL). Table 4 shows input variables.

**Figure 1:**
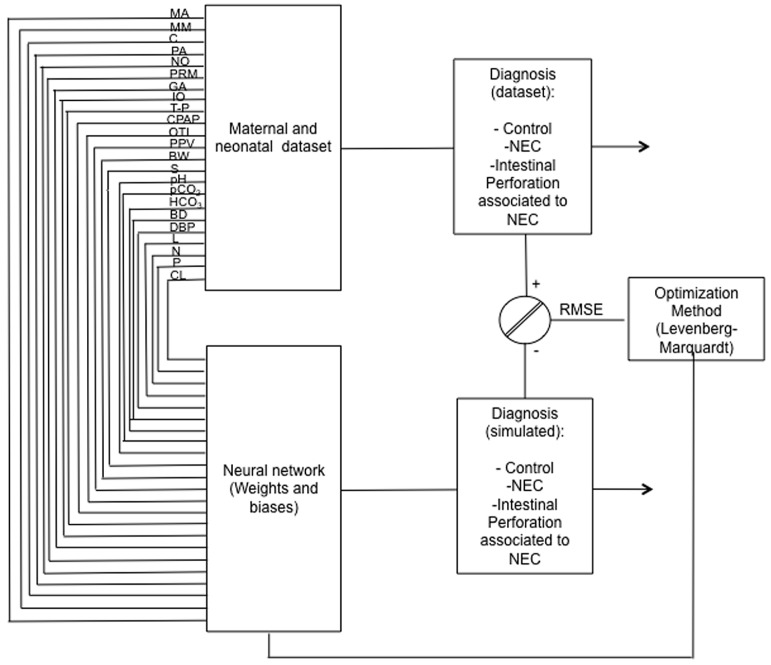
A representative network architecture of Intestinal perforation (IP) model. The learning procedure used by ANN for the estimation of IP associated with NEC from 23 maternal and neonatal variables at birth (maternal age, maternal morbidity, chorioamnionitis, prenatal antibiotic, number of offsprings, premature rupture of membranes, gestational age, oxygen therapy (indirect oxygen, T-piece, continuous positive airway pressure, positive pressure ventilation cycles, orotracheal intubation), birth weight, sex, arterial pH, blood gas (CO_2_, HCO_3_, base deficit), diastolic arterial blood pressure, number of total leukocytes, neutrophils, platelets and catheter location), trained by the Levenberg-Marquardt optimization algorithm. The same architecture was used for IP estimation with birth and hospitalization variables.

A representative ANN architecture for intestinal perforation associated with NEC model is depicted in Figure 1, with 23 input variables at birth and 1 output variable: diagnosis (No NEC, NEC or IP associated with NEC).

#### 2.3.3. Statistical Test for ANN Model Validation

We applied a statistical test (slope and intercept test [31]) in which the upper and lower intervals of the slope and intercept from linear regression models of the experimental database versus the simulated ones (learning and validation database) must approach 1.0 and 0, respectively; with a 99.8% confidence level according to the Student *T*-test.

The regression coefficient (*R*^2^) was then obtained from linear regression models for each ANN model:

 Simulation=a+b exp 

### 2.4. Sensitivity Analysis

In order to identify key factors that play an important role in predicting intestinal perforation associated with NEC, we performed a sensitivity analysis to the trained and validated neural network, as previously described ([26,27] and Garson algorithm in Appendix B), allowing to determine which input variables (maternal and neonatal parameters) are more important (or sensible) to attain precise output values (diagnosis).

## 3. Results

The aim of this study was to obtain ANN models estimating intestinal perforation associated with NEC (IP) in order to differentiate from NEC or no NEC (Control) diagnosis and to investigate key factors for the prediction. For this purpose, extracted data from maternal and neonatal records were used to train two BPNN models: (1) an IP model with birth variables or (2) an IP ANN model with birth and hospitalization parameters. Such design will allow to explore the importance of risk factors at birth compared to hospitalization parameters, obtained from a sensitivity analysis of both models.

All neonates with IP associated with NEC diagnosis in NICU (*N* = 27) were chosen while NEC (*N* = 23) or control groups (*N* = 27) were carefully selected in order to have similar gestational ages in all groups. Neonatal birth weight was significantly higher in the control group (1384 ± 95.5 g) compared to NEC or IP groups (1085 ± 65.8 g and 1141 ± 65.21 g, respectively; see Table 2, *p* < 0.05).

**Figure 2:**
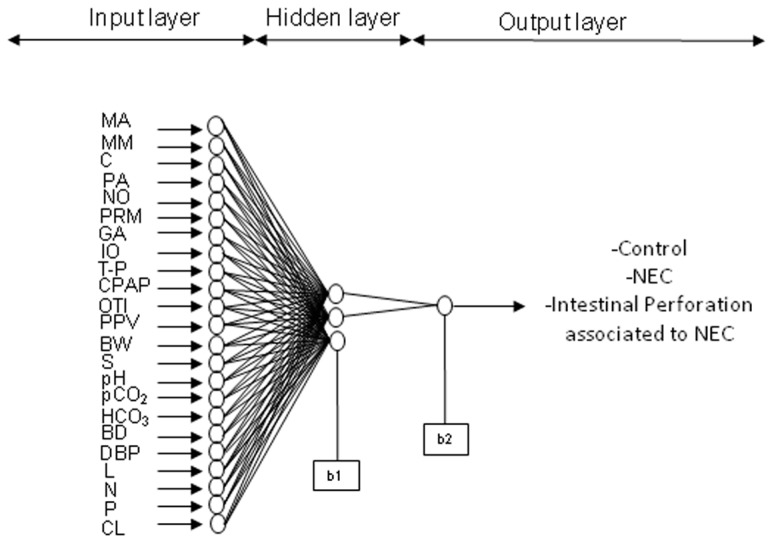
Intestinal perforation associated with NEC ANN model. Representative estimation model for the diagnosis of intestinal perforation associated with NEC (IP) with the 23 input variables described in Figure 1, 3 neurons in the hidden layer (b1) and 1 neuron in the output layer (b2).

For both models, distinct parameters (number of neurons) and transfer functions were tested, finding the best performance to be the hyperbolic tangential function (TANSIG) in the hidden layer and the Log-sigmoid function (LOGSIG) in the output layer. For both models, 30,000 runs (with 1000 epochs) were applied in the hidden layer (from one neuron to two or three neurons). For the ANN model predicting IP at birth, the final architecture was 23 input variables, 3 neurons in the hidden layer, and 1 neuron in the output layer (IP associated with NEC, NEC or no NEC diagnosis): 23-3-1; while the final topology for the ANN IP model at birth and during hospitalization was 35-2-1. The representative neural architecture for the estimation of IP diagnosis is depicted in Figure 2, while the equations, weights, and biases of both models are reported in Appendix B and Appendix C (Equations (A1)–(A6) and Table A1 and Table A2).

**Figure 3:**
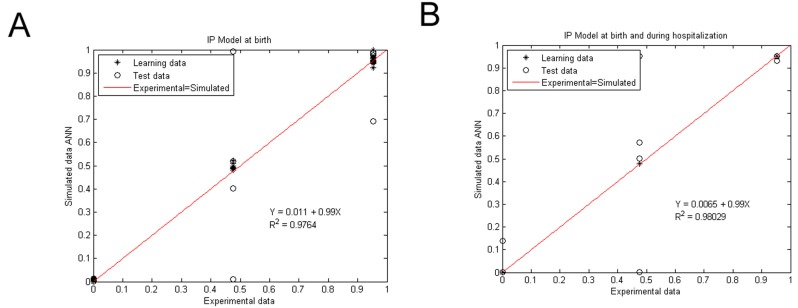
Experimental vs. ANN-simulated values for IP diagnosis estimation. Scatter plot of IP model at birth (**A**) and at birth and during hospitalization (**B**). Red lines indicate the linear regression model on scatter points. Open circles and closed diamonds show experimental and learning data, respectively.

Both intestinal perforation associated with NEC models had a good accuracy, with regression coefficients of *R*^2^ = 0.9764 and *R*^2^ = 0.98029 for the IP model with birth variables (Figure 3A) and the IP model with birth and hospitalization parameters (Figure 3B), respectively, evaluated by the linear regression between the experimental and simulated data, as well as the statistical tests from these plots, with a 99.8% confidence level for all determinations (Table A3 and Table A4 in Appendix C).

**Figure 4:**
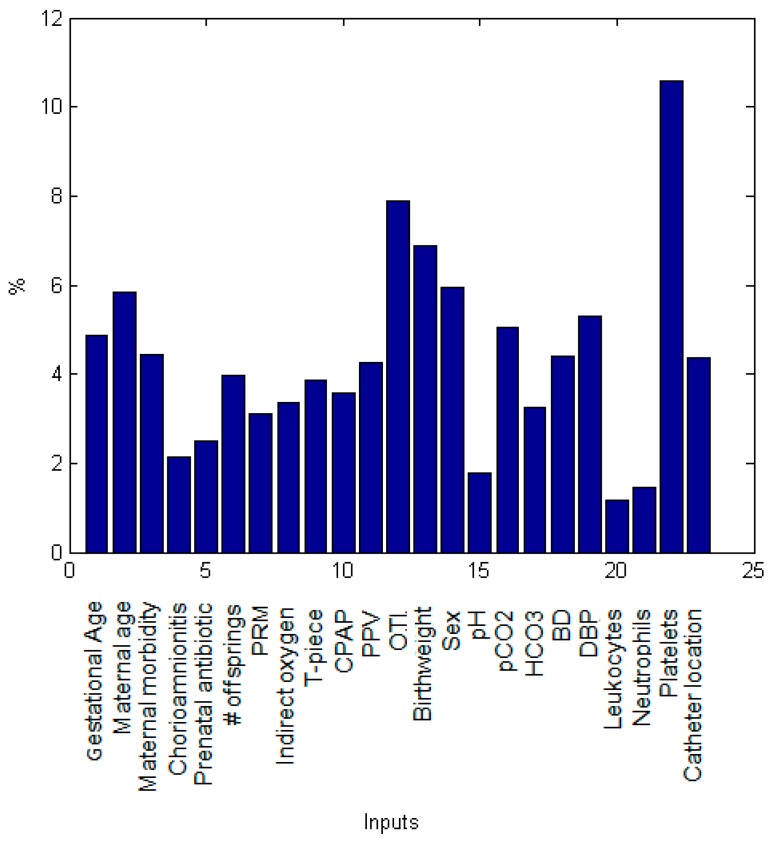
Relative contribution of each predictor variable to the estimation for the IP ANN model at birth. The relative influence histogram shows the mathematical importance of each predictor variable in the model evaluated by a sensitivity analysis. It is measured as a percentage of quantitative significance on the Y-axis for each predictor parameter at birth.

**Figure 5:**
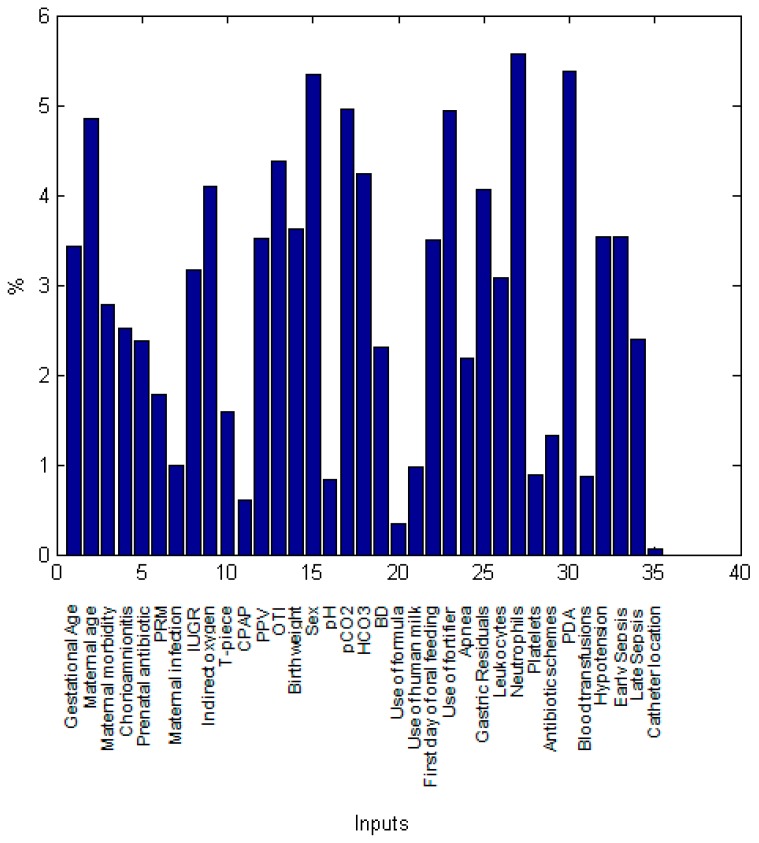
Relative contribution of each predictor variable to the estimation for the IP ANN model at birth and hospitalization. The relative influence histogram shows the mathematical importance of each predictor variable in the model evaluated by a sensitivity analysis. It is measured as a percentage of quantitative significance on the Y-axis for each predictor parameter at birth and during hospitalization.

In order to identify which maternal and neonatal factors were critical for the prediction of intestinal perforation associated with NEC in both models, we performed a sensitivity analysis which depicts the importance of individual factors (inputs variables) in the modeling of the IP diagnosis (Figure 4 and Figure 5). At birth, neonatal platelets number was the most significant parameter for IP prediction followed by the use of orotracheal intubation (OTI) as oxygen therapy, birthweight, sex, maternal age, neonatal diastolic blood pressure (DBP), pCO_2_ and gestational age (Figure 4).

In the IP ANN model with birth and hospitalization parameters, the number of neonatal neutrophils, PDA, sex, pCO_2_, use of fortifier, maternal age, OTI, HCO_3_, indirect oxygen, and gastric residuals were the key factors with the highest relative contribution to the estimation of intestinal perforation. These variables were followed by birthweight, PPV, maternal age, first day of oral feeding, hypotension and early sepsis (Figure 5). Overall, maternal factors accounted for 18.1% of the importance in estimating IP while neonatal birth variables were responsible for 44.4% (oxygen therapy 14%, arterial blood gas and laboratory findings 30.4%) and hospitalization factors for 37.5%.

## 4. Discussion

An emphasis in forecasting intestinal perforation associated with NEC from NEC alone was the objective of this work, which was attained with good performances by both models (at birth or at birth and during hospitalization), the regression coefficients between the experimental and predicted data were *R*^2^ > 0.97. Learning to estimate perforation depends on all variables from individual cases working together in a multidimensional process in order to obtain a pattern of forecasting, and allowing for the non-linear relations between variables to be determined during the learning process, make these ANN models highly valuable for clinicians since prediction approaches personalized medicine.

Identification of critical factors and the assessment of how output changes by varying input variable values one by one, added knowledge in the field and will permit additional understanding of risk factors for the prediction of a future intestinal perforation associated with NEC. Previously unreported key variables for the prediction of IP in both models were: orotracheal intubation, arterial blood gas parameters (pCO_2_ and HCO_3_), use of milk fortifier and maternal age. Therefore, attention should be paid to these parameters.

The relative contribution of each predictor variable also allowed to verify that the models are doing what they are intended to do (estimation of intestinal perforation diagnosis), by finding variables involving literature-known factors, that may allow an early diagnosis and follow-up of premature neonates. With respect to previously described risk factors for intestinal perforation associated with NEC, lower birth weight [32,33,34], decreased gestational age [32,33], apnea episode [32], presence of sepsis [32], lower platelet count [6,32,35] were also obtained by our models. Risk factors contained in the final GutCheck model included gestational age, packed red blood cells transfusion, unit NEC rate, late-onset sepsis, multiple infections, hypotension treated with inotropic medications, Black or Hispanic race, birth in a different NICU and metabolic acidosis [13]. As well, risk parameters in a disease progression statistical regression model comprise gender, gestational age, and birth weight [13,36].

In the matched prospective multicenter cohort study by Berkhout et al., multivariable logistic regression modeling demonstrated only 2 independent variables to be associated with an increased risk of NEC: administration of predominantly formula feeding and the cumulative number of parenteral feeding days. Remarkably, administration of any antibiotics initiated within 24 h after birth was associated with a reduced risk of NEC [37].

We found that male gender was a highly predictive parameter for intestinal perforation associated with NEC compared to only NEC. There are only a few studies where male gender has been significantly associated to increased risk of NEC [36,38] or not [39]. Duci et al. reported that gender was not statistically significant when comparing patients with NEC treated medically vs. NEC requiring surgery [39,40]. However, more work is needed to conclude that males are more likely to progress to intestinal perforation. To include a greater number of patients from multicenter studies could clarify this.

### 4.1. Maternal Burden

In regard to maternal factors, older age (>38 years) followed by preeclampsia or hypertension determined perforation by NEC in the ANN models. In contrast, Lee et al. reported a lack of association between preeclampsia and NEC, demonstrating that neutrophil-to-lymphocyte ratio (NLR) at the time of admission and multiparity was associated with the occurrence of NEC [41]. Zhang et al. did not find a difference in perinatal factors including hypertensive disorders, diabetes mellitus, intrahepatic cholestasis, heart disease, hypothyroidism, premature rupture of membranes, placental abruption, antenatal steroid use or mode of delivery between NEC and control groups [41,42]. The literature, however, evaluating the association between maternal preeclampsia and neonatal NEC is conflicting. Bashiri et al. reported that maternal hypertensive disorders may be independent predictors of NEC in children smaller than 1500 g at birth [43]. Other studies have demonstrated that the risk of NEC is increased by intrauterine growth restriction and maternal smoking [44].

Recent studies performed in twins have suggested that a genetic variation in an intergenic region of chromosome 8, labeled as the “NECRISK” region may be associated with increased risk for surgical NEC. Although no specific genes have been identified, pathway analyses have indicated possible pathways related to growth factor, calcium, and G-protein signaling, and others associated with inflammation that may contribute to NEC complications [45].

### 4.2. First Day of Life

From laboratory findings, a higher neutrophil count and lower platelet numbers predicted perforation by the models. In this regard, thrombocytopenia has been associated with NEC and perforation in several studies [6].

In our study, arterial blood gases (pCO_2_ and HCO_3_) were the most important variables to predict IP associated with NEC. It is known that a high base deficit from umbilical cord arterial samples at birth can contribute to NEC in growth-restricted infants [6,46]. In our models, metabolic acidosis combined with higher total numbers of leukocytes, neutrophils, and platelets forecasted intestinal perforation with NEC. In agreement with our data, a study by Duci and colleagues reported a statistically significant difference in pH between patients with medically treated NEC vs. NEC requiring surgery (7.35 vs. 7.2, *p* < 0.0001) and identified a lower risk for surgery in patients with a later onset of NEC and higher pH values [40]. Altogether, these findings support the need for a critical care and follow-up in the first hours of life as a predictive event for developing intestinal perforation associated with NEC.

### 4.3. During Hospitalization

Regarding variables taken from hospitalization data, the presence of PDA, the use of fortifier, early-onset sepsis, hypotension, and gastric residuals were the most important factors for estimating intestinal perforation. Except for the use of fortifier, all other variables have been associated with NEC but not specifically to intestinal perforation [34]. The use of fortifier is a new variable for IP prediction and will be part of the hypothesis to be tested in a future study examining patients. Tepas proposed seven criteria that may be considered as predictive of surgical intervention: bandemia, positive blood culture, acidosis, hypotension, thrombocytopenia, hyponatremia, or neutropenia [47], and some of these parameters were also shown as relevant in the models performed in this study.

It is also important to describe the less/non-predictive factors such as catheter location, formula feeding, and CPAP. At birth, catheter location seemed a valuable parameter, however when taking into account birth and hospitalization data together, its contribution diminished compared to other key factors. With respect to formula feeding, the NICU has implemented the use of donor milk when the patient’s mother is not available, perhaps explaining why the use of bovine formula was not an important variable in this study.

### 4.4. Limitations and Strengths of the Study

We have to acknowledge the limitation of this work to be the relatively small dataset size (*n* = 76) however, the use of anthropometric, clinical and laboratory findings as well as a balanced dataset gave accurate results. Another important limitation is the fact that it is a single center study nonetheless, our institution is a tertiary care hospital that concentrates complicated pregnancies from all over the country. We also have to recall that both ANN models predict the diagnosis of intestinal perforation associated with NEC within the limits of the variables range (Table 3 and Table 4). Also, adding more variables to the model (birth and hospitalization data) did not improve the power of prediction. 

The strengths of this work include a complete maternal and patient information from variables at birth and associated with the course of the disease taking into account lifestyle, morbidity, anthropometric, clinical, enteral feeding, mechanical ventilation, blood gas, medications and laboratory findings of the study population from three balanced groups. We report both key factors as well as less-important factors for the prediction of IP. Learning by ANN to estimate a pattern of intestinal perforation associated with NEC from individual cases relied on several variable parameters, making such tools highly valuable for clinical setting since they allow a more precise prediction of the outcome.

## 5. Conclusions

Both BPNN models were able to accurately estimate intestinal perforation associated with NEC. Furthermore, key maternal and neonatal variables were found by the models involving well-known factors reported in the literature, as well as new parameters that may allow the early diagnosis and follow-up of premature neonates at risk of surgical NEC. Our results highlight the value of integrating maternal and neonatal variables at birth and during hospitalization variables in BPNN models to better estimate surgical NEC. A suggestion for new modeling will be to incorporate data from birth, day 3 and day 7, as well as from multi-center studies. We hope our models may be useful to preselect at-risk patients for perforation associated with NEC before randomization for a strict follow-up that could result in different surgical interventions.

## Figures and Tables

**Table 1:** Clinical and demographic characteristics of the maternal population.

Variables	Control Group (*n* = 27)	NEC (*n* = 23)	IP (*n* = 26)	*P*
Age (years)	28.04 ± 1.77	25.74 ± 1.60	31.31 ± 1.41	0.06
Preeclampsia (%)	14.81	30.43	26.92	0.39
Hypertension (%)	7.407	8.63	11.54	0.87
Overweight/Obesity (%)	11.11	0	11.54	0.24
Hypothyroidism (%)	18.52	4.34	3.84	0.11
Chorioamnionitis (%)	11.11	17.39	11.54	0.77
No. Offsprings (range)	1–3	1–2	1–3	
Intra Uterine Growth Restriction (%)	11.11	39.13	26.92	0.07

Obesity defined as a Body mass index (BMI) greater or equal to 30 (World Health Organization). Chorioamnionitis was defined as an acute inflammation or infection of the membranes and chorion of the placenta.

**Table 2:** Clinical and demographic characteristics of the neonatal population.

Variables	Control (*n* = 27)	NEC (*n* = 23)	IP (*n* = 26)	*P*
Gestational age (weeks)	30.8 ± 0.42	30.3 ± 0.52	30.3 ± 0.46	0.71
Birthweight (g)	1384 ± 95.5	1085 ± 65.8	1141 ± 65.21	0.01 ^1^
Height (cm)	39.76 ± 0.82	36.72 ± 0.77	37.29 ± 0.89	0.02 ^1^
Sex (male, %)	59.26	43.48	57.71	0.48

^1^*P* < 0.05 between Control and NEC groups.

**Table 3:** Input range conditions in the IP ANN model at birth.

Input Variable	Range
Gestational age (weeks)	25–34.4
Maternal age (years)	14–44
Maternal morbidity	0–15 (see Appendix A)
Chorioaminionitis (y/n)	0–1
Prenatal antibiotic (y/n)	0–1
Number of offsprings	0–3
Premature rupture of membranes (y/n)	0–1
Indirect oxygen (y/n)	0–1
T-piece (y/n)	0–1
CPAP (y/n)	0–1
PPV (y/n)	0–1
OTI (y/n)	0–1
Birth weight (g)	560–3125
Sex (female/male, 1/2)	1–2
Arterial pH value	6.96–7.41
Arterial CO_2_ (mm Hg)	19.4–72.1
Arterial HCO_3_ (mmol/L)	9.6–34.5
Arterial Base Deficit (mEq/L)	−16.9–7.9
Diastolic arterial blood pressure	20–56
Leukocytes (cells/mm^3^)	2800–39,940
Neutrophils (cells/mm^3^)	1008–24,000
Platelets (cells/mm^3^)	12,200–439,000
Catheter location (absence, 0; high or low placed umbilical arterial, 1 or 2)	0–2

**Table 4:** Input range conditions in the IP ANN model at birth and during hospitalization.

Input Variable	Range
Gestational age (weeks)	25–34.4
Maternal Age (years)	14–44
Maternal morbidity	0–15 (see Appendix A)
Chorioaminionitis (y/n)	0–1
Prenatal antibiotic (y/n)	0–1
Premature rupture of membranes (y/n)	0–1
Maternal infection (y/n)	0–1
IUGR (y/n)	0–1
Indirect oxygen (y/n)	0–1
T-piece (y/n)	0–1
CPAP (y/n)	0–1
PVV (y/n)	0–1
OTI (y/n)	0–1
Birth weight (g)	560–3125
Sex (female/male, 1/2)	1–2
Arterial pH value	6.96–7.41
Arterial CO_2_ (mm Hg)	19.4–72.1
Arterial HCO_3_ (mmol/L)	9.6–34.5
Arterial Base Deficit (mEq/L)	−16.9–7.9
Use of formula (y/n)	0–1
Use of human milk (y/n)	0–1
First day of oral feeding (day)	1–3
Apnea (y/n)	0–1
Gastric residuals (y/n)	0–1
Diastolic arterial blood pressure	20–56
Leukocytes (cells/mm^3^)	2800–39,940
Neutrophils (cells/mm^3^)	1008–24,000
Platelets (cells/mm^3^)	12,200–439,000
Catheter location (absence, low, high, hepatic)	0–3
Antibiotic schemes	1–2
PDA (y/n)	0–1
Blood transfusions (y/n)	0–1
Hypotension (y/n)	0–1
Early sepsis (y/n)	0–1
Late sepsis (y/n)	0–1
Catheter location (absence, 0; high or low placed umbilical arterial, 1 or 2)	0–2

## Appendix A

List of Maternal morbidities classification for the ANN models:

1: Preeclampsia
2: Hypertension
3: Gestational Diabetes Mellitus
4: Overweight
5: Adolescence
6: Hypothyroidism
7: Gestational Diabetes Mellitus and Hypothyroidism
8: Type II Diabetes and preeclampsia and overweight and hypertension
9: Polycystic ovary
10: Cervicovaginitis
11: Gestational Diabetes Mellitus and Preeclampsia
12: Preeclampsia and overweight
13: Hypothyroidism and overweight
14: Diabetes and candidiasis
15: Preeclampsia and carbohydrate intolerance

## Appendix B

In the hidden and output layers, each neuron (*n*) has coefficients assigned to them, termed weights (*Wi* and *Wo*) and biases (*b*_1_ and *b*_2_) such as:

*n*_1_ = *Wi* × *In*_1_ + *Wi* × *In*_2_ +, …, + *Wi* × *In*_k_ + *b*_1_ (A1)

where *In* is the input variable. The value of each neuron is the argument of the transfer functions (*f* and *g*):

IP, NEC or no NEC (output) = *g*(*Wo* × *f* (*Wi* × *In* + *b*_1_) + *b*_2_) (A2)

*f* is a hyperbolic tangent transfer function (TANSIG) and *g* is a linear transfer function (PURELIN) or Log-Sigmoid function (LOGSIG).

We applied different transfer functions in both ANN models and the best performance was found with TANSIG-LOGSIG therefore, Equation (A2) is now Equation (A3):

Where *n_output_* is:

(A3) $$output=\frac{1}{\left(1+\exp\left(-n_{output\_layer}\right)\right)}\;\;n_{output\_layer}=\underset{s}{\sum }\left\{Wo_{\left(l,s\right)}.\left[\frac{2}{1+e^{-2.\left(\sum_{k}Wi_{\left(s,k\right)}.In_{k}+b1_{\left(s,1\right)}\right)}}-1\right]\right\}+b2_{\left(l,1\right)}\;$$

ANN learning

In this work, to change the weights and biases, we applied the Levenberg-Marquardt algorithm, following our previously reported methods [26,27]. This uses the adaptation as follows:

(A4) $$\Delta w={\left(J^{T}J+\mu I\right)}^{-1}J^{T}e\;$$

where:

*J* is the Jacobian matrix (first derivative)
*e* is a vector of network errors
*μ* is the combination coefficient with a value of 0.001
*I* is the identity matrix.

The Root Mean Square Error (*RMSE*) was applied as the error function which describes the performance of the network according to the following equation Equation (A5):

(A5) $$RMSE=\sqrt{\frac{\left(\sum_{q=1}^{Q}{\left(y_{q,exp}-y_{q,ANNsim}\right)}^{2}\right)}{Q}}\;$$

where:

*Q* is the number of data points (*n* = 76),
$y_{q,exp}$ is the experimental data,
$y_{q,ANNsim}$ is the network prediction.

Results for intestinal perforation ANN models

The obtained ANN models for diagnosis of Intestinal Perforation (IP) associated with NEC followed equation Equation (A6) with TANSIG-LOGSIG:

*n_output_* is:

(A6) $$\;\mathrm{IP}\_\mathrm{or}\_\mathrm{NEC}\_\mathrm{or}\_\mathrm{no}\_\mathrm{NEC}=\frac{1}{\left(1+exp\left(-n_{output\_layer}\right)\right)}\;\;n_{output\_layer}=\underset{s}{\sum }\left\{Wo_{\left(l,s\right)}.\left[\frac{2}{1+e^{-2.\left(\sum_{k}Wi_{\left(s,k\right)}.In_{k}+b1_{\left(s,1\right)}\right)}}-1\right]\right\}+b2_{\left(l,1\right)}\;$$

Equation (A6) estimates intestinal perforation associated with NEC, with weights and biases from Table A1 (IP ANN model at birth, 23-3-1) and Table A2 (IP ANN model at birth and during hospitalization, 35-2-1) in Appendix C.

Sensitivity analysis

To obtain the relative importance of variables in predicting intestinal perforation associated with NEC, we performed a sensitivity analysis based on the partitioning of connection weights proposed by Garson in Equation (A7):

(A7) $$\;I_{j}=\frac{\sum_{m=1}^{Nh}\left(\left(\frac{\left|W\;{\;}_{jm}^{\;ih}\right|}{\sum_{k=1}^{Ni}\left|W\;{\;}_{km}^{\;\;ih}\right|}\right)\times \;\left|W\;{\;}_{mn}^{\;ho}\right|\right)}{\sum_{k=1}^{Ni}\left\{\sum_{m=1}^{Nh}\left(\frac{\left|W\;{\;}_{km}^{\;ih}\right|}{\sum_{k=1}^{Ni}\left|W\;{\;}_{km}^{\;ih}\right|}\right)\times \;\left|W\;{\;}_{mn}^{ho}\right|\right\}}\;$$

where:

$I_{j}$ is the relative importance of the jth input variable on the output variable,
Ni is the number of input neurons,
Nh is the number of hidden neurons,
W is the connection weight,
And the superscripts i, h and o refer to input, hidden and output layer.

## Appendix C

**Table A1:** Weights and biases for the IP ANN model at birth (3 neurons in the hidden layer, k = 3 and l = 1).

	Wi_{s,k}_		
Wi_{s,1}_	1.3267125	3.1584473	−1.0502622
Wi_{s,2}_	−1.7146594	−1.8984633	2.2922617
Wi_{s,3}_	4.8419826	0.346469	0.172989
Wi_{s,4}_	0.7706828	−1.8081623	−0.1177451
Wi_{s,5}_	−0.6644504	−1.4773511	0.641453
Wi_{s,6}_	1.7169406	1.6965454	−0.980111
Wi_{s,7}_	1.4663105	0.5359049	1.1481214
Wi_{s,8}_	0.5342111	3.4738356	0.2333177
Wi_{s,9}_	2.5968973	1.9983119	−0.1671108
Wi_{s,10}_	0.0638118	0.9084672	−2.1722897
Wi_{s,11}_	−0.4195773	2.0502619	−1.7919031
Wi_{s,12}_	1.5347326	−1.6982282	4.0750083
Wi_{s,13}_	3.4133979	−3.0526489	−1.3469327
Wi_{s,14}_	1.7925231	1.4563156	−2.5644274
Wi_{s,15}_	0.5678812	−0.7150829	−0.5980426
Wi_{s,16}_	2.661324	2.4458136	−0.7857453
Wi_{s,17}_	0.0671341	0.8060811	−1.9744531
Wi_{s,18}_	−3.7567751	0.823349	−0.531168
Wi_{s,19}_	−4.0508192	1.2459655	−0.7937825
Wi_{s,20}_	−0.8384736	0.3953479	0.1355509
Wi_{s,21}_	−1.5220229	0.0202477	−0.1580771
Wi_{s,22}_	−4.7012854	−5.0669983	−2.2215755
Wi_{s,23}_	−2.8142496	−0.4138948	1.3408794
	Wo_{1,1}_	Wo_{1,2}_	Wo_{1,3}_
Wo_{l,s}_	−5.5289631	4.3969459	5.5399183
b1_{23,1}_	b1_{s,1}_		
	1.6391183		
	−2.673269		
	5.3597672		
	b2_{l,s}_		
b2_{1,1}_	−0.2889819		

**Table A2:** Weights and biases for the IP ANN model at birth and during hospitalization (2 neurons in the hidden layer, k = 2 and l = 1).

	Wi_{s,k}_	
Wi_{s,1}_	−0.3751959	−7.3554209
Wi_{s,2}_	8.7757177	0.8013817
Wi_{s,3}_	3.8650996	1.8292029
Wi_{s,4}_	−3.5645456	−1.5785954
Wi_{s,5}_	1.6843183	3.4310907
Wi_{s,6}_	−1.0314863	−2.8521103
Wi_{s,7}_	1.7744987	−0.1751799
Wi_{s,8}_	−6.0887131	−0.1231969
Wi_{s,9}_	2.9294796	−5.9005369
Wi_{s,10}_	−1.6834391	1.6662543
Wi_{s,11}_	−0.2871787	−1.0537841
Wi_{s,12}_	−1.7927934	−5.9023226
Wi_{s,13}_	8.3022116	−0.2731127
Wi_{s,14}_	−2.7479431	5.0231616
Wi_{s,15}_	−5.5854112	5.6349576
Wi_{s,16}_	1.2288445	0.4765211
Wi_{s,17}_	−4.9111278	−5.5446961
Wi_{s,18}_	−6.0798957	2.5347047
Wi_{s,19}_	−2.9004902	−1.8560627
Wi_{s,20}_	−0.3563622	0.3671644
Wi_{s,21}_	0.4792034	−1.6491173
Wi_{s,22}_	−0.7248806	7.1122093
Wi_{s,23}_	−0.7406803	10.360829
Wi_{s,24}_	0.1160807	4.8359502
Wi_{s,25}_	0.4699811	−8.6634359
Wi_{s,26}_	2.1030666	−4.5441764
Wi_{s,27}_	3.309815	8.8156683
Wi_{s,28}_	0.6025175	1.3123462
Wi_{s,29}_	2.1301649	−0.5153287
Wi_{s,30}_	4.0466436	−7.5138313
Wi_{s,31}_	−1.4889536	0.2544955
Wi_{s,32}_	2.287042	−5.3644162
Wi_{s,33}_	4.4242002	−2.8862814
Wi_{s,34}_	1.6291247	−3.5560135
Wi_{s,35}_	0.0894362	−0.0343897
	Wo_{1,1}_	Wo_{1,2}_
Wo_{l,s}_	7.952817	−9.1921023
b1_{35,1}_	b1_{s,1}_	
	2.3093149 1.7504724	
	b2_{l,s}_	
b2_{1,1}_	−14.149185	

**Table A3:** Slope and intercept values for the statistical test of the IP ANN model at birth.

Birth Variables	
a_lower_	a_upper_
−0.0395	0.0607
b_lower_	b_upper_
0.9074	1.0703

**Table A4:** Slope and intercept values for the statistical test of the IP ANN model at birth and during hospitalization.

Birth and Hospitalization Variables	
a_lower_	a_upper_
−0.0394	0.0524
b_lower_	b_upper_
0.9191	1.0683
